# Human tissue kallikrein 14 induces the expression of IL‐6, IL‐8, and CXCL1 in skin fibroblasts through protease‐activated receptor 1 signaling

**DOI:** 10.1111/febs.70170

**Published:** 2025-07-07

**Authors:** Laura Sasiadek, Ewa Bielecka, Katherine Falkowski, Magdalena Kulczycka, Grzegorz Bereta, Anna Maksylewicz, Natalia Zubrzycka, Ewelina Dobosz, Joanna Kozieł, Justyna Drukała, Maciej Lech, Natalia Horbach, Marcin Poręba, Klaudia Brix, Grzegorz Dubin, Jan Potempa, Tomasz Kantyka

**Affiliations:** ^1^ Malopolska Centre of Biotechnology Jagiellonian University Krakow Poland; ^2^ Faculty of Biochemistry, Biophysics and Biotechnology Jagiellonian University Krakow Poland; ^3^ Doctoral School of Exact and Natural Sciences Jagiellonian University Krakow Poland; ^4^ Department of Medicine IV LMU University Hospital, LMU Munich München Germany; ^5^ Faculty of Chemistry Wroclaw University of Science and Technology Poland; ^6^ School of Science Constructor University Bremen Germany; ^7^ Department of Oral Immunity and Infectious Diseases, School of Dentistry University of Louisville KY USA

**Keywords:** C‐X‐C chemokine ligand‐1 (CXCL1), fibroblast, interleukin‐6 (IL‐6), interleukin‐8 (IL‐8), kallikrein (KLK), protease‐activated receptor‐1 (PAR‐1), wound healing

## Abstract

Human tissue kallikrein 14 (KLK14) is protease with trypsin/chymotrypsin specificity that is abundant in the skin. It is involved in skin desquamation and wound healing by cleaving cell–cell adhesion molecules and extracellular matrix components. In the process of wound healing, a paracrine communication between the epithelium, human skin fibroblasts (HSFs), and immune cells is essential for proper regulation. Previous reports highlighted stimulation of interleukin‐6 (IL‐6), interleukin‐8 (IL‐8), and growth‐regulated alpha protein (CXCL1) production by keratinocyte‐conditioned medium in fibroblast cells and implicated these cytokines in cancer. Here, we hypothesize that KLK14 may be a paracrine mediator released by keratinocytes that activates fibroblasts via proteinase‐activated receptor (PAR) pathway, affecting the HSF secretome. Semiquantitative real‐time PCR and ELISA demonstrated that proteolytically active KLK14 induced the expression of *IL‐6*, *IL‐8*, and *CXCL1* by 15‐, 847‐, and 50‐fold, respectively, and resulted in the release of the proteins in ng/ml quantities from stimulated HSFs to the culture medium. Through the implementation of the PAR‐1 antagonist RWJ 56110, we demonstrated that the KLK14‐mediated release of IL‐6 and IL‐8 is dependent on PAR‐1 activation. Contrarily, PAR‐1 activation was shown to function as a limiting factor in the KLK14‐mediated CXCL1‐releasing pathway. Furthermore, human recombinant IL‐6, IL‐8, and CXCL1 enhanced closure of a cell‐free gap in an HaCaT cell monolayer, mimicking wound healing of keratinocytes in the skin. KLK14‐stimulated HSF conditioned media also induced wound healing in the HaCaT model in an IL‐6‐dependent manner, as a cytokine‐neutralizing antibody significantly decreased this activity. Thus, KLK14 in the skin may participate in paracrine signaling between fibroblasts and keratinocytes, in that keratinocyte‐secreted KLK14 initiates the release of IL‐6, IL‐8, and CXCL1 from fibroblasts, which in turn act on proximal keratinocytes to trigger their migration for wound closure. Our findings add to the understanding of the role of KLK14 in the related processes of wound healing and tumor development.

## Graphical abstract

Human tissue kallikrein 14 (KLK14), a skin protease, may mediate paracrine signaling in wound healing. KLK14 released by epithelial cells activates fibroblasts via PAR‐1 activation, inducing IL‐6, IL‐8, and CXCL1 expression. These cytokines in turn promote keratinocyte proliferation and migration, resulting in wound closure. Conditioned media from KLK14‐stimulated fibroblasts mimicked this effect, especially via IL‐6. Our findings suggest that KLK14 regulates skin repair and may be linked to tumorigenesis through fibroblast–keratinocyte communication.

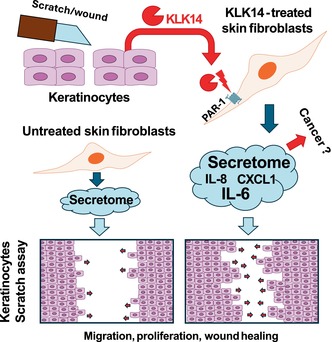

**Abbreviations**
ADAM: A disintegrin and metalloproteinase domain‐containing protein
CXCL: C‐X‐C motif chemokine ligand
DMEM: Dulbecco's modified Eagle medium
ECM: extracellular matrix
EF2: elongation factor 2
EGF: epithelial growth factor
ELISA: enzyme‐linked immunosorbent assay
EMI: epithelial–mesenchymal interaction
ERK: extracellular signal‐regulated kinase
FaDu: human cell line from a hypopharyngeal carcinoma
FBS: fetal bovine serum
GRO: growth‐regulated oncogene
H.I.: heat inactivated
HaCaT: immortal keratinocyte cell line from human skin
IL: interleukin
KLK: kallikrein
LEKTI: Lympho‐epithelial Kazal‐type‐related inhibitor
MAPK: mitogen‐activated protein kinase
MMP: matrix metalloproteinase
PAR: protease‐activated receptor
PBS: phosphate‐buffered saline
PCR: polymerase chain reaction
RFU: relative fluorescence units
rhCXCL1: human recombinant CXCL1
rhEGF: human recombinant EGF
rhIL‐6: human recombinant IL‐6
rhIL‐8: human recombinant IL‐8
RT‐qPCR: reverse transcription‐semi quantitative real‐time PCR
SEM: standard error of measurement
SPINK: serine protease inhibitor Kazal‐Type
TGF: transforming growth factor
TMPRSS2: transmembrane serine protease 2
TNF: tumor necrosis factor

## Introduction

The skin is the largest organ in the human body responsible for protecting the host from mechanical damage and external environmental factors such as pathogens and UV radiation [1, 2]. The epidermis mostly consists of keratinocytes that are generated from proliferating stem cells in the *Stratum basale* and migrate towards the outermost layer, the *Stratum corneum*, undergoing gradual differentiation and cell death [3]. The underlying dermis functions as a support and cushions the body from external mechanical impact. It is predominantly composed of fibroblasts that produce the connective tissue and extracellular matrix proteins such as collagens and laminin that also make up the basement membrane which separates the dermis from the epidermis [4].

Human tissue kallikreins are a family of 15 serine proteases (KLK1‐KLK15) with either trypsin‐ or chymotrypsin‐like activity [5]. They present the largest continuous gene cluster in the human genome and are located on chromosomal locus 19q13.3–13.4 [6]. Tissue kallikreins are scattered throughout the body but possess a substantial role in the human skin where their proteolytic activity is required for the maintenance of normal skin physiology [7, 8]. KLK5‐7 and KLK14 are responsible for the degradation of corneodesmosomal components resulting in skin desquamation required for the shedding of squames [9, 10].

KLKs are produced by keratinocytes mainly in the *Stratum granulosum*, but they have been shown to diffuse to other areas in the skin including the dermis [7, 8, 11, 12]. Their expression and activation are tightly controlled, and dysregulation leading to a KLK system imbalance has been linked to skin damage. Atopic dermatitis involves the overexpression of KLK7, and elevated levels of KLK6 and KLK8 contribute to psoriasis. At the same time, a loss of function mutation in the *SPINK5* gene encoding a KLK inhibitor, results in overactivation of KLK5 and KLK7. In turn, this leads to skin barrier dysfunction in Netherton syndrome [13, 14, 15]. KLKs are crucial mediators in cutaneous wound healing where they contribute to extracellular matrix remodeling. KLK6 and KLK14 cleave ECM glycoproteins, adhesion molecules, and collagens; KLK6 may activate the sheddase ADAM10; KLK4, KLK5, and KLK8 may activate metalloproteinase meprin β; and KLK14 may activate membrane type matrix metalloproteinases, all important events contributing to cell migration and re‐epithelialization [16, 17, 18, 19].

KLKs, including KLK14, were implicated in protease‐activated receptor (PAR)‐mediated signaling. PARs are a family of four (PAR1‐4) G protein‐coupled receptors, in which proteolytic processing by an external protease (canonically thrombin and/or trypsin) reveals the ligand sequence, which then binds and activates the receptor. PAR receptors are involved in cell activation and inflammatory reaction, and their activation was implicated in cancer [20]. KLK‐family peptidases, including KLK14, were found to activate the PAR‐2 receptor in skin‐derived epithelial cells [21], in HEK cells expressing the PAR receptors [22]. In parallel, KLK14 and PAR2 staining was correlated in the tissue samples from inflammatory skin disorder patients [21]. KLK14 expression also was found on the protein level in 16 gastric cancer cell lines and in human colon adenocarcinomas, while KLK14‐mediated processing of PAR‐2 was identified in the HT29 gastric cancer cell line [23]. What's more, recently we have shown that KLK14 may induce neutrophil extracellular traps release (NETosis) in a PAR‐2‐dependent manner [24].

It has been demonstrated previously that skin keratinocytes and fibroblast cells participate in the reciprocal paracrine signaling, which affects both the transcriptome and secretome of fibroblasts, which in turn induce metabolic changes in keratinocytes [25]. Interestingly, this study identified IL‐6, IL‐8, and CXCL‐1, all involved in the inflammatory and regenerative response, among the most upregulated genes induced in fibroblast cells treated with epithelial secretome and in the clinical tumor samples, correlating the wound healing response with cancer. We therefore hypothesized that this secretome‐mediated stimulation of HSF response by epithelial cells may be mediated by proteolytic activation of PAR‐family receptors by KLK14.

The research presented in this paper pinpoints the role of KLK14 in wound healing through its ability to modify the skin fibroblast secretome by upregulating the expression and secretion of IL‐6, IL‐8 and CXCL1. These three important factors essential in wound healing were stimulated by KLK14 in a PAR‐1 and PAR‐3‐dependent manner. Here we show that these three factors individually promote keratinocyte wound healing, and through a paracrine‐signaling event, the factors secreted from fibroblasts may affect neighboring keratinocytes to initiate migration for wound closure.

## Results

### KLK14 augments the IL‐6, IL‐8, and CXCL1 secretion and gene expression in HSF

**Fig. 1:**
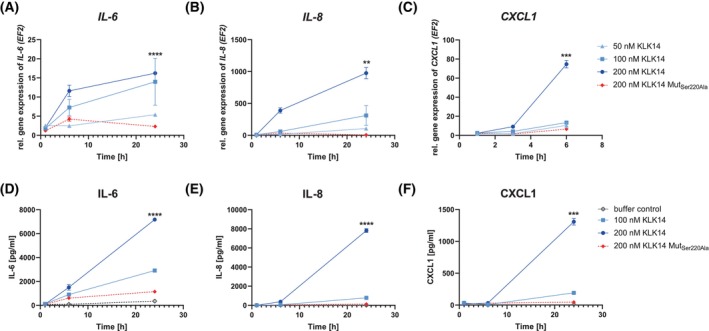
KLK14 induces increased expression and secretion of IL‐6, IL‐8, and CXCL1 in human primary skin fibroblast cells (HSF). Effects of KLK14 on *IL‐6*, *IL‐8*, and *CXCL1* gene expression (A–C) and protein secretion (D–F) in HSF. Cells were stimulated with KLK14 or inactive KLK14 Mut_Ser220Ala_, for indicated times (A–F). Conditioned media were collected, and mRNA was extracted from the cell lysates. Real‐time quantitative polymerase chain reaction results were calculated using the ΔΔCT method, normalized to the *EF2* housekeeping gene, cells treated with buffer alone were used as a control (A–C). Protein release was quantified using enzyme‐linked immunosorbent assay (D–F). The results are presented as mean ± SEM of 4 (A, B, D, F), 2–4 (C), 4–8 (E) replicates. The statistical analysis of the difference between HSF stimulated with 200 nm KLK14 compared to the vehicle control (buffer control) after 24 h (*CXCL1* expression after 6 h) was calculated using an unpaired *t*‐test using Welch's correction: **P ≤ 0.01, ***P ≤ 0.001, ****P ≤ 0.0001.

KLK14's effects on the secretome of HSF were assessed by studying *IL‐6*, *IL‐8*, and *CXCL1* gene expression and secretion utilizing the RT‐qPCR and ELISA, respectively. HSF treatment with KLK14 enhanced cytokine/chemokine expression and their secretion into conditioned media in a time‐ and concentration‐dependent manner (Fig. 1A–F). Specifically, 24 h stimulation with 200 nm KLK14 significantly increased the expression and secretion of IL‐6 and IL‐8 (Fig. 1A,B,D,E). These 15‐ and 847‐folds of enhanced expressions of the *IL‐6* and *IL‐8* genes (Fig. 1A,B), respectively, are correlated with 17‐ and 547‐fold increases of IL‐6 and IL‐8 secretion (Fig. 1D,E) as evident from the measured cytokine concentrations. In the case of CXCL1, the 50‐fold increased expression of the *CXCL1* gene after 6 h stimulation with 200 nm KLK14 corresponded to a 91‐fold increase in chemokine secretion by HSF at 24 h post‐treatment (Fig. 1C,F). Notably, the replacement of the active form of KLK14 with the catalytically incompetent KLK14 variant, KLK14 Mut_Ser220Ala_, resulted in no changes in *IL‐6*, *IL‐8*, and *CXCL1* gene expression (Fig. 1A,B,C) and cytokine/chemokines release into the conditioned media (Fig. 1D,E,F), as compared to HSF treated with the vehicle control. In addition, the effect of native KLK14 was compared to the heat‐inactivated KLK14. Neither of the inactive variants were capable of inducing IL‐6, IL‐8, nor CXCL1 secretion to the culture medium (data not shown). This unambiguously indicates the importance of KLK14's proteolytic activity for the stimulation of the expression and secretion of biologically active mediators by HSF.

### PAR‐1 activation is responsible for KLK14‐mediated IL‐6 release in HSF

**Fig. 2:**
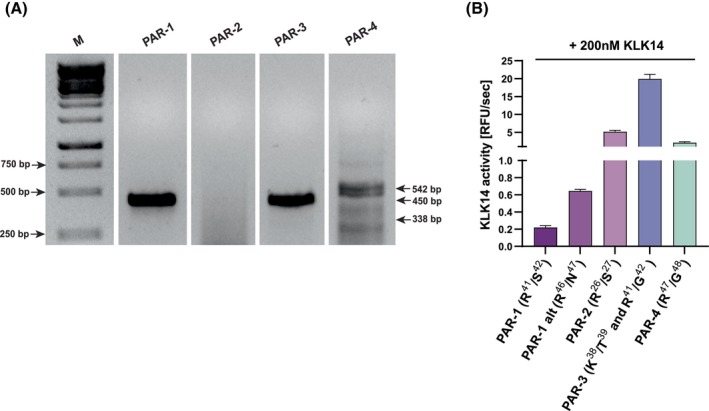
Proteinase‐activated receptors (PARs) gene expression pattern in human skin fibroblasts (HSF) and the ability of KLK14 for recognition of PAR activation sequences. (A) Real‐time polymerase chain reaction was performed on mRNA isolated from HSF cells, gene products were visualized using agarose gel electrophoresis, and the gene transcripts were observed at their expected sizes: 431 bp for *PAR‐1*, 443 bp for *PAR‐3*, and 542 bp for *PAR‐4*. The lacking *PAR‐2* gene product would be visualized at 338 bp. (B) KLK14 cleavage of PAR activation sequences. Fluorogenic substrates (10 μm) representing activation sequences of PAR receptors (with the expected cleavage site marked by an arrow): PAR‐1 (LDPR↓SFLL), PAR‐1 alternative (GFLLR↓NPND), PAR‐2 (SKGR↓SLIG), PAR‐3 (LPIKTFR↓G); and PAR‐4 (PAPR↓GYPG) were added to 200 nm KLK14, and a kinetic fluorometric measurement was performed at an excitation at 350 nm and emission at 460 nm. The resulting RFU/s values were presented as mean ± SEM from three independent experiments (*n* = 10).

It was shown that in primary lung fibroblast cultures, IL‐6 is released upon PAR‐1 activation [26]. Therefore, in the skin context, we investigated if KLK14‐mediated IL‐6 release in HSF is a result of similar PAR processing. First, we confirmed the expression of *PAR* mRNA in HSF. To this end, total mRNA was isolated from cell lysates of unstimulated HSF, and *PAR*‐specific transcripts were amplified by RT‐PCR. Amplicons were resolved by agarose gel electrophoresis yielding a band pattern that revealed the presence of *PAR‐1*, *PAR‐3*, and *PAR‐4* gene transcripts but not of the *PAR‐2* gene. This indicates a lack of *PAR‐2* expression in HSF under normal culture conditions (Fig. 2A). Thus, to assess if KLK14 may activate PARs, we performed a kinetic fluorometric assay measuring KLK14‐mediated processing of fluorescent substrates based on the PAR activating sequences, encompassing the canonical (for PAR‐1 to PAR‐4) and alternative (PAR‐1 alternative) cleavage sites. KLK14 was able to cleave all substrates consisting of all PAR‐1, PAR‐2, PAR‐3, and PAR‐4 activation sequences, of which the PAR‐3 substrate was cleaved most efficiently (Fig. 2B). Due to the presence of both arginine and lysine residues in the PAR3 substrate sequence, we used LC–MS to determine the KLK14 hydrolysis location. This allowed us to identify Arg^41^‐Gly^42^ in the PAR‐3 substrate as the preferred site, in accordance with KLK14 substrate specificity.

**Fig. 3:**
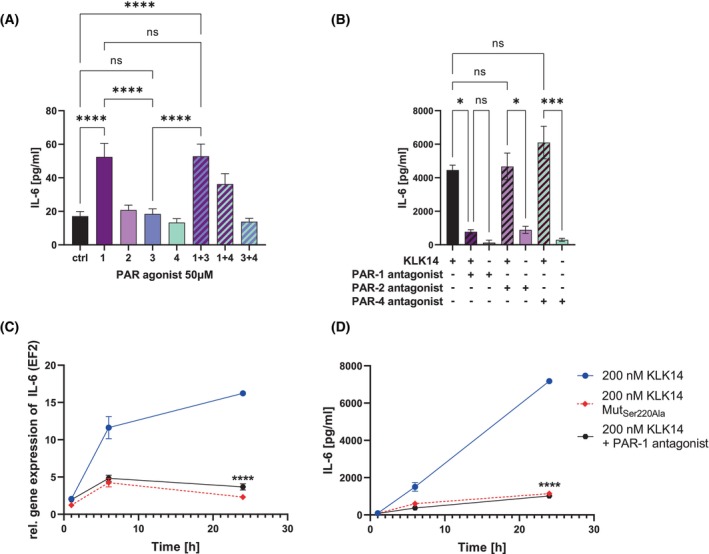
KLK14 activates proteinase‐activated receptor 1 (PAR‐1) resulting in expression of the *IL‐6* gene and enhanced secretion of the cytokine by human skin fibroblasts (HSF). (A) HSF were stimulated with 50 μm PAR‐1, PAR‐2, PAR‐3, or PAR‐4 agonist, or their specified mixes for 24 h, and IL‐6 levels were measured in conditioned media. (B) HSF were pre‐incubated with 50 μm PAR‐1, ‐2 or ‐4 antagonist, respectively, for 1 h, followed by a stimulation with 200 nm KLK14 for 6 h, and the level of IL‐6 was measured in the conditioned media. (C, D) HSF were pre‐stimulated with 50 μm PAR‐1 antagonist for 1 h and then incubated with 200 nm KLK14 up to 24 h. The conditioned media were collected, and mRNA was extracted from cell lysates. (C) Real‐time quantitative polymerase chain reaction was conducted, and the results were calculated using the ΔΔCT method, normalized to the *EF2* housekeeping gene, cells treated with buffer alone were used as a control. (D) Protein release was quantified using a hIL‐6 enzyme‐linked immunosorbent assay. The statistical analysis of the difference of the gene expression (C) or the protein concentration in conditioned media (D) after 24 h of HSF stimulation with 200 nm KLK14 and the PAR‐1 antagonist compared to HSF stimulated with only 200 nm KLK14. The results are presented as mean ± SEM of 10 (A), 2 (B), 4 replicates (C, D). The statistical analysis was calculated using Welch's t‐test (C, D) or a one‐way ANOVA test (A, B): *P ≤ 0.05, ***P ≤ 0.001, ****P ≤ 0.0001, ns, not significant. Statistics is shown only for the selected samples.

To observe whether IL‐6 gene expression and secretion in HSF depends on PAR activation, cells were stimulated with the PAR1‐4 agonists (TFLLR‐NH_2_, SLIGRL‐NH_2_, H‐TFRGAP‐OH, or AY‐NH_2_, respectively) or their specified combinations. The conditioned media were collected after 24 h, and ELISA was performed. Results illustrate that only the PAR‐1 agonist was able to induce IL‐6 secretion in the HSF culture, and no combination further enhanced this effect (Fig. 3A). These findings support the notion that KLK14‐mediated IL‐6 release in HSF depends solely on KLK14's ability to activate PAR‐1. To further verify this finding, HSF cells were pre‐stimulated for 1 h with the PAR‐1, PAR‐2, or PAR‐4 antagonist (RWJ 56110, FSLLRY‐NH2, ML 354, respectively), prior to KLK14 stimulation. Indeed, out of these signaling inhibitors, only the PAR‐1 antagonist blocked the KLK14‐mediated IL‐6 secretion (Fig. 3B). This indicates that the IL‐6 release is a direct result of PAR‐1 activation by KLK14.

To further characterize the observed effect, the KLK14‐mediated IL‐6 stimulation was followed in a time‐dependent manner. The RT‐qPCR analysis and hIL‐6 ELISA results demonstrated that in the presence of the PAR‐1 antagonist, KLK14‐mediated *IL‐6* gene expression (Fig. 3C) and the cytokine secretion were both significantly reduced in a time‐dependent manner (Fig. 3D). The KLK14‐mediated increase in the *IL‐6* gene expression was 2.5‐fold lower at 6 h and 4.5‐fold lower at 24 h when PAR‐1 activation was blocked in comparison to the KLK14‐stimulated sample in the absence of PAR‐1 antagonist (Fig. 3C). KLK14‐mediated IL‐6 release was also affected, as the IL‐6 culture medium levels were 3‐fold lower at 3 h, 4‐fold lower at 6 h, and 7‐fold lower at 24 h in the presence of PAR‐1 antagonist (Fig. 3D). When PAR‐1 was blocked, the KLK14‐mediated increase in the *IL‐6* gene expression was similar to the effect observed for the catalytically incompetent KLK14 Mut_Ser220Ala_, indicating that inhibition of PAR‐1 signaling completely blocks the effect of KLK14 proteolytic activity on primary HSF. Therefore, these findings suggest that the KLK14‐mediated IL‐6 expression and secretion pathway is directly dependent on the PAR‐1 activation by KLK14.

### KLK14‐mediated PAR‐1 activation enhances IL‐8 production in HSF

**Fig. 4:**
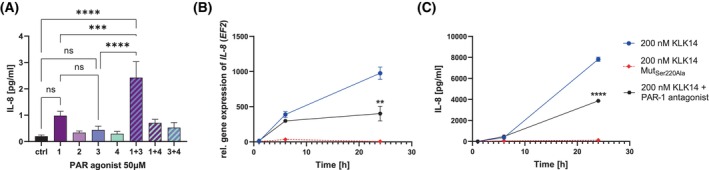
KLK14 activation of proteinase‐activated receptor 1 (PAR‐1) partially contributes to the enhanced *IL‐8* gene expression and secretion of the cytokine by human skin fibroblasts (HSF). (A) HSF were stimulated with 50 μm PAR‐1, PAR‐2, PAR‐3, or PAR‐4 agonist, or their specified mixes for 24 h, and cytokine levels in conditioned media were measured. (B, C) HSF were pre‐stimulated with 50 μm PAR‐1 antagonist for 1 h and then incubated with 200 nm KLK14 for up to 24 h. The conditioned media were collected, and mRNA was extracted from cell lysates. (B) Real‐time quantitative polymerase chain reaction was conducted, and the results were calculated using the ΔΔCT method, normalized to the *EF2* housekeeping gene; cells treated with buffer alone were used as a control. (A, C) Protein release was quantified using a hIL‐8 enzyme‐linked immunosorbent assay. The statistical analysis presents the difference in gene expression (B) or the protein concentration in conditioned media (C) after 24 h of HSF stimulation with 200 nm KLK14 and the PAR‐1 antagonist compared to HSF stimulated with only 200 nm KLK14. The results are presented as mean ± SEM of 10 (A), 4 (B), 4–8 (C) replicates. The statistical analysis was calculated using Welch's t‐test (B, C) or a one‐way ANOVA test (A): **P ≤ 0.01, ***P ≤ 0.001, ****P ≤ 0.0001, ns, not significant. Statistics is shown only for the selected samples.

To characterize the KLK14‐mediated *IL‐8* gene expression pathway and the chemokine secretion, we attempted to assess if PAR‐1 activation is also involved in this context. Therefore, the PAR activation effect on *IL‐8* gene expression and IL‐8 release was analyzed in a manner similar to IL‐6 analysis. The RT‐qPCR and IL‐8 ELISA results revealed a likely involvement of PAR‐1 in KLK14‐mediated IL‐8 release. Secretion of IL‐8 was increased upon stimulation with the PAR‐1 agonist, and the synergistic effect was observed for stimulation with the mixture of the PAR‐1 and PAR‐3 agonist. Nevertheless, the secretion levels of this cytokine were very low (Fig. 4A). KLK14 was an effective inducer of IL‐8 production in primary skin fibroblast culture (Fig. 4B,C). *IL‐8* gene expression was induced already after 3 h of KLK14 incubation and increased further until 24 h. The presence of the PAR‐1 antagonist limited this stimulation to some extent, especially at longer incubation times, yet still, ~40% of induction could be observed after 24 h of incubation. In contrast, the inactive catalytic mutant of KLK14 did not induce IL‐8 expression significantly, confirming that KLK14 proteolytic activity is indispensable for this stimulation (Fig. 4B). In agreement with the gene expression data, ELISA of the culture supernatants yielded similar results. Again, the levels of IL‐8 were increased by KLK14 stimulation and the effect was dependent on the proteolytic activity of the enzyme, as KLK14 Mut_Ser220Ala_ did not induce any observable effect. The presence of the PAR‐1 antagonist during KLK14 stimulation only partially reduced IL‐8 levels in the culture medium, as concentrations of about half of the ones detected in the KLK14‐treated HSF culture were still observed (Fig. 4C). Together, these results indicate that while proteolysis by KLK14 is necessary, the KLK14 effect on *IL‐8* expression and IL‐8 medium secretion cannot be fully explained only by PAR‐1 activation and suggest that likely an additional KLK14 signaling pathway is involved. Indeed, the results of the concurrent stimulation of HSF with the PAR‐1 and PAR‐3 agonist indicate that both PAR‐1 and PAR‐3 activation could be at least partially responsible for the increase of IL‐8 production.

### KLK14‐mediated PAR‐1 activation limits the production of CXCL1 in HSF

**Fig. 5:**
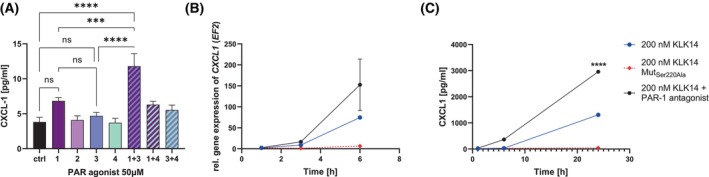
Proteinase‐activated receptor 1 (PAR‐1) functions as the limiting mechanism in the KLK14‐mediated CXCL1 release and gene expression in human skin fibroblasts (HSF). (A) HSF were stimulated with 50 μm PAR‐1, PAR‐2, PAR‐3, or PAR‐4 agonist, or their specified mixes, for 24 h, and conditioned media were collected. (B, C) HSF were pre‐stimulated with 50 μm PAR‐1 antagonist for 1 h and then incubated with 200 nm KLK14 for up to 24 h. The conditioned media were collected and mRNA was extracted from cell lysates. (B) Real‐time quantitative polymerase chain reaction was conducted, and the results were calculated using the ΔΔCT method, normalized to the *EF2* housekeeping gene, cells treated with buffer alone were used as a control. (A, C) Protein release was quantified using a hCXCL1 enzyme‐linked immunosorbent assay. The statistical analysis presents the difference in protein concentration in conditioned media (C) after 24 h of HSF stimulation with 200 nm KLK14 and the PAR‐1 antagonist compared to HSF stimulated with only 200 nm KLK14. The results are presented as mean ± SEM of 19 (A) and 4 replicates (B, C). The statistical analysis was calculated using Welch's t‐test (B, C) or a one‐way ANOVA test (A): ***P ≤ 0.001, ****P ≤ 0.0001, ns, not significant. Statistics is shown only for the selected samples.

Since PAR‐1 was involved in the KLK14‐mediated IL‐6 and IL‐8 increase in gene expression and medium release, we aimed to characterize the mechanism of KLK14‐induced CXCL1 production. We again observed the increase in CXCL1 levels upon PAR‐1 agonist treatment; however, the effect was weak and did not reach the statistical significance. Interestingly, the combination of PAR‐1 + PAR‐3 agonists resulted in the marked increase in CXCL1 secretion, indicating a potential PAR‐3 role in the CXCL1 stimulation in HSF culture (Fig. 5A). When analyzing the effect of KLK14 on the production of CXCL1 in primary human fibroblast culture, again, the proteolytic activity of KLK14 was fundamental for the induction of *CXCL1* gene expression and CXCL1 production, as the catalytically inactive KLK14 variant did not elicit any effect. To our surprise, however, stimulation of the cell culture with KLK14 in the presence of the PAR‐1 antagonist resulted in a markedly higher response, when compared to KLK14 treatment alone. *CXCL1* gene expression and medium secretion were both approximately 2‐fold higher after 6 h and 24 h of stimulation, respectively, compared to the HSF culture incubated with catalytically incompetent KLK14 (Fig. 5B,C). These results suggest that in primary HSF culture, activation of PAR‐1 functions rather as the mechanism limiting the CXCL1 release, not a CXCL1 production inducer, while other protease‐activated mediators may be responsible for the CXCL1 increase.

### IL‐6, IL‐8, and CXCL1 affect wound healing in HaCaT model

**Fig. 6:**
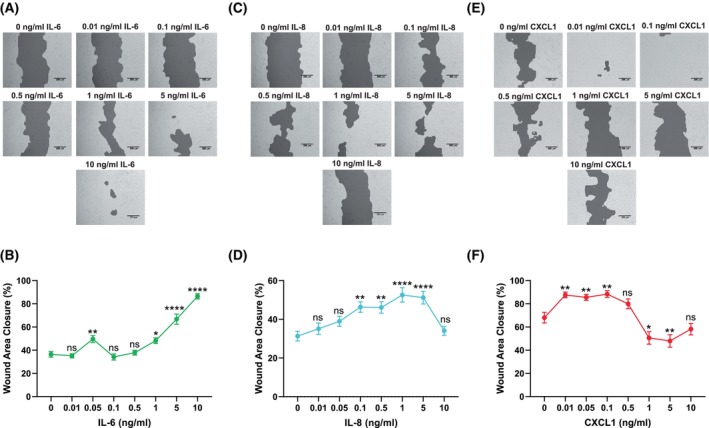
Effects of human recombinant IL‐6, IL‐8, and CXCL1 (rhIL‐6, rhIL‐8, rhCXCL1) on HaCaT *in vitro* wound healing. HaCaT were grown between the chambers of Ibidi culture inserts on 24‐well cell culture plates. When cell confluency was reached, the culture inserts were removed and the cells were supplemented with rhIL‐6, rhIL‐8 or rhCXCL1 for 72 h. Images were taken using bright field microscopy and a 10× objective lens at 72 h. The images shown include the TScratch cell‐free area mask used for wound area closure calculations. For the mask‐free images, please refer to the supporting data (please see Data availability statement for details; (A), C, E). The wound area closure in each respective image was measured and presented as the percentage of the unstimulated control from incubation time‐point 0 h (B, (D), F). The results are presented as mean ± SEM of 16–30 replicate images and compared to the unstimulated control after 72 h. The statistical analysis was calculated using a one‐way ANOVA test: *P ≤ 0.05, **P ≤ 0.01, ****P ≤ 0.0001.

Fibroblasts and keratinocytes are adjacent cells in the skin that communicate reciprocally through paracrine signaling. Therefore, to assess if HSF‐derived IL‐6, IL‐8, and CXCL1 affect keratinocyte migration and proliferation, *in vitro* wound healing assays were performed utilizing HaCaT keratinocytes that were grown in the presence of culture inserts. When confluency was reached, the inserts were removed and the cells were stimulated for 72 h with increasing concentrations of human recombinant IL‐6 (rhIL‐6), human recombinant IL‐8 (rhIL‐8), or human recombinant CXCL1 (rhCXCL1), respectively. Images of the corresponding wells were taken using bright field microscopy, and the wound area closure was measured (Fig. 6A,C,E). The unstimulated control at time 0 h was regarded as 100% of the wound area for normalization, and the percentage of the wound area closure was calculated for each image (Fig. 6B,D,F).

HaCaT wound healing was induced by rhIL‐6, rhIL‐8, and rhCXCL1 in a concentration‐dependent manner. The 0.01, 0.1, and 0.5 ng·mL^−1^ of rhIL‐6 did not show any effect on HaCaT wound closure. At higher 1–10 ng·mL^−1^ IL‐6 concentrations, the wound area decreased linearly after 72 h when compared to unstimulated cells, indicating that IL‐6 effectively contributes to the wound healing response in HaCaT culture.

Further, the results demonstrated that similarly to IL‐6, rhIL‐8 at concentrations of 0.01 and 0.05 ng·mL^−1^ had no significant effect on HaCaT wound healing, when compared to the unstimulated control. A moderate increase in wound healing was observed for 0.1–5 ng·mL^−1^ IL‐8 in a concentration‐dependent manner. Then, 10 ng·mL^−1^ rhIL‐8 did not affect the wound healing, indicating the roughly bell‐shaped response curve.

In contrast, the low concentrations of CXCL1 (0.01–0.1 ng·mL^−1^) were able to significantly enhance wound healing in the HaCaT culture. Then, 0.5 ng·mL^−1^ of rhCXCL1 displayed no significant effect on HaCaT wound healing, while high concentrations 1–10 ng·mL^−1^ of CXCL1 even inhibited the wound healing response, as indicated by significantly larger remaining wound area, compared to the untreated control. Again, the CXCL1 response was characterized by the bell‐shaped curve, similar, though more prominent as for IL‐8.

### KLK14‐stimulated HSF conditioned media improves wound healing in HaCaT model

**Fig. 7:**
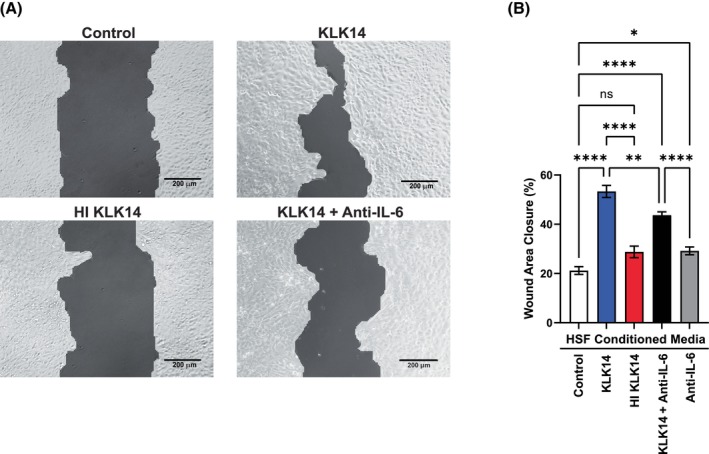
Effects of KLK14‐stimulated human skin fibroblasts (HSF) conditioned media transfer on HaCaT *in vitro* wound healing. HSF were grown until confluency on 24‐well plates and were stimulated with 200 nm KLK14 or 200 nm HI (heat inactivated) KLK14 in DMEM for 24 h, and the conditioned media were collected. Subsequently, HaCaT were grown between the chambers of Ibidi culture inserts on 24‐well plates until confluency was reached. The culture inserts were removed, the cells were rinsed with PBS and were stimulated with 50% of unstimulated HSF conditioned media or HSF conditioned media +100 ng·mL^−1^ anti‐IL‐6 or unstimulated HSF conditioned media +100 ng·mL^−1^ anti‐IL6, all with 5 μm KLK14 inhibitor for 48 h. (A) Images were taken of respective wells using bright field microscopy and a 10× objective lens. The images shown include the TScratch cell‐free area mask used for wound area closure calculations. For the mask‐free images, please refer to the supporting data (please see Data availability statement for details). (B) The wound area closure from each image was measured and was presented as the percentage of the unstimulated control at 0 h. The results were graphed as mean ± SEM of 19–24 replicate images and compared to the unstimulated control from 48 h. The statistical analysis was calculated using an ordinary one‐way ANOVA test: *P ≤ 0.05, **P ≤ 0.01, ****P ≤ 0.0001, ns, not significant.

Keratinocytes and fibroblasts compose adjacent, but functionally different layers of the skin, which both participate in the effective wound closure. We therefore aimed to investigate if KLK14‐induced HSF excretions will affect the wound healing response of keratinocytes. To that end, the HSF cultures were stimulated for 24 h with 200 nm KLK14, heat‐inactivated (HI) KLK14, or with the buffer as the negative vehicle control. Then, cell supernatants were collected, diluted with fresh DMEM to 50% and applied on the HaCaT cultures in the wound healing model. In addition, to evaluate the effect of KLK14‐induced HSF IL‐6 secretion on HaCaT wound healing, a series of samples with 50% KLK14‐conditioned media was supplemented with 100 ng·mL^−1^ of neutralizing anti‐IL6 antibody. Images of each respective well were taken (Fig. 7A), and the wound area closure from each respective image was measured and was presented as the percentage of the unstimulated control at 0 h (Fig. 7B).

KLK14‐stimulated HSF conditioned media increased wound healing, when compared to the unstimulated control nearly 3‐fold. This effect was significantly decreased in the presence of the IL‐6 neutralizing antibody, indicating an increase in IL‐6 levels in the KLK14‐stimulated HSF medium as an important factor enhancing wound healing in the HaCaT culture. Importantly, conditioned media from the HSF culture treated with heat‐inactivated KLK14 did not increase wound healing signifying that KLK14 proteolytic activity is required to induce this effect. These results suggest that KLK14 may participate in wound healing through paracrine signaling between the fibroblasts and keratinocytes in the skin.

## Discussion

Tissue kallikreins are important mediators in the process of cutaneous wound healing. They are expressed predominantly in the *Stratum granulosum*, where they induce keratinocyte migration and proliferation [7, 12, 27]. Yet, upon wounding, they penetrate through the sub‐lining layers of the epithelium into the dermal portion of the skin. There, their proteolytic activity is essential for wound remodeling through their ability to cleave extracellular matrix proteins [7].

KLK14, found in the stratum corneum, accounts for a significant portion of the epidermal trypsin‐like activity. It cleaves adhesion proteins and extracellular matrix components, contributing to wound healing [9, 11, 28, 29]. This study demonstrates that KLK14 also influences wound healing by modulating the secretion of signaling molecules, such as IL‐6, IL‐8, and CXCL1, from skin fibroblasts, which are crucial for paracrine signaling between fibroblasts and keratinocytes.

Our research focused on the effects of KLK14 in the regulation of three important factors involved in wound healing: IL‐6, IL‐8, and CXCL1. Upon wounding of the skin, IL‐6 is known to promote collagen production by fibroblasts, induce angiogenesis, and promote the M1 to M2 macrophage phenotype switch that concludes the inflammatory stage of wound healing [30, 31, 32, 33]. The chemokines IL‐8 and CXCL1 function as neutrophil chemoattractants, promote keratinocyte proliferation and migration, and likewise induce angiogenesis [34].

KLK14 increases the release of IL‐6, IL‐8, and CXCL1 from HSF in a time‐ and concentration‐dependent manner. The involvement of KLK14's proteolytic activity in cytokine release was confirmed through the use of catalytically inactive KLK14 mutants. Additionally, the activation of protease‐activated receptors (PARs), particularly PAR‐1, is implicated in cytokine induction, though alternative pathways, including PAR‐3 activation, may also contribute. In each case, both gene expression and conditioned media levels were enhanced with prolonged time and increasing concentrations of KLK14 from 50 up to 200 nm. Although a concentration of 200 nm KLK14 may seem high for a secreted enzyme, KLKs have been shown to not only be freely diffusing but also localized, forming confined concentration gradients. For example, KLK14 and KLK4 are found in membrane protrusions colocalized with hepsin and TMPRSS2, which may lead to the local increase in KLK14 concentration near the cell surface [35] The physiological concentrations of secreted KLK14 in skin are difficult to estimate, with some reports hinting at 10–40 nm [7, 8], while at the same time, KLK expression and extracellular activity may be significantly increased upon cell stimulation. For example, a stimulated induction of KLK14 expression reached nearly 3000‐fold upon primary normal human epidermal keratinocyte treatment with *S. aureus‐*conditioned medium [36], a finding potentially relevant for atopic dermatitis. Our data indicate robust cellular response already at 50 nm KLK14, at least for IL‐6 stimulation, suggesting the concentration dependence approaching physiological levels of KLK14 in the tissue. This concentration range is in line with the previous reports, where minimum of 30 nm KLK14 was required to trigger the PAR‐2 activation‐induced calcium flux and 100 nm and above concentrations were used for further experiments in an *in vitro* cell model [23]. Here we show that KLK14 was able to release greater amounts of IL‐6 and IL‐8 than CXCL1 in HSF. This agrees with the observation that low levels of CXCL1 are sufficient to induce wound healing, while concentrations exceeding 0.5 ng·mL^−1^ regress wound closure as demonstrated in our HaCaT wound model. Indeed, we demonstrated that different levels of IL‐6, IL‐8, and CXCL1 are necessary for the proper wound healing of HaCaT *in vitro*. The degree of wound closure gradually increased with increasing concentrations of IL‐6, and the cytokine healing ability was maximal at relatively high concentrations. Contrarily, IL‐8 and CXCL1 demonstrated a bell‐shaped response curve, both promoting and inhibiting wound closure depending on the available chemokine concentration.

Upon stimulation of HSF with the catalytically incompetent KLK14 active site mutant as well as a heat‐inactivated/denatured KLK14, it was evident that the mechanism involved in the KLK14‐mediated release of IL‐6, IL‐8, and CXCL1 into the conditioned media was dependent on KLK14's proteolytic activity. Therefore, we investigated if the activation of PARs was responsible for the induction of IL‐6, IL‐8, and CXCL1 gene expression and secretion. PARs are receptors that require N‐terminal proteolytic processing to induce their activation resulting in intracellular signal transduction. In addition, activated PARs may dimerize with unprocessed PARs leading to their transactivation [37]. Importantly, PARs have previously been described to be involved in wound healing processes [37, 38, 39]. For example, PAR‐1 activation is known to induce keratinocyte proliferation and regulates immune responses by inducing the migration of eosinophils [38, 40]. Notably, it has been demonstrated that PARs can be activated by members of the kallikrein family of proteases. KLK4‐6 and KLK14 have been previously shown to cleave PAR‐1 at the canonical R41‐S42 extracellular cleavage site and KLK14 may also cleave PAR‐1 at the R46‐N47 position [22, 41]. Here, we have confirmed the expression of *PAR‐1*, *PAR‐3*, and *PAR‐4*, but not *PAR‐2* in primary human skin fibroblasts. This observation is consistent with the previous reports. PAR‐2 transcripts were detected in human keratinocytes, but only in trace amounts in human fibroblasts and trypsin was only a weak PAR agonist in fibroblasts [42]. Furthermore, human keratinocytes were found to be responsive to PAR‐2 agonists, tryptase and trypsin, while the response of dermal fibroblast was consistent with the PAR‐1‐dependent signaling [43]. Here we report that KLK14 has the ability to cleave all the PAR activation sequences, as indicated by fluorescent substrate analysis. KLK14 can process both the canonical and alternative site in the PAR‐1 activation sequence, in agreement with the above‐mentioned KLK4‐6 and 14; however, the alternate site sequence was hydrolyzed more efficiently in comparison to the canonical one. Yet, to our surprise, these sequences are processed by KLK14 rather slowly, especially when compared to KLK14 hydrolysis of PAR‐2, PAR‐3, and PAR‐4 sequences *in vitro*. This confirms the previous reports of KLK14/PAR‐1 processing, obtained on short peptide sequences analyzed by HPLC [22], but contrasts our cell‐derived data. Here PAR‐1 inhibition using the receptor antagonist resulted in the near‐complete inhibition of IL‐6 and significant limitation of IL‐8 production. This intriguing discrepancy may be explained by cell membrane sequestration of KLK14. Indeed, KLK14 presence was previously identified as a part of the prostate‐cancer‐related protease network, where secreted KLK14 and KLK4 are recruited to membrane protrusions decorated with cell surface‐bound hepsin and TMPRSS2 [35]. It was recently demonstrated that similar recruitment of thrombin by thrombomodulin expressed on the endothelial cells shifts the PAR‐1 signaling of thrombin from the canonical site (Arg41) to the alternate Arg46 location and results in the enhanced kinetics and cytoprotective signaling [44]. Interestingly, the partial effect of PAR‐1 inhibition on the KLK14‐induced IL‐8 production and the surprising increase of CXCL1 levels in the presence of the PAR‐1 antagonist indicate that another proteolysis‐sensitive pathway is involved in the regulation of these cytokines. Based on the identified substrate specificity of KLK14, on the HSF response to the PAR agonist panel and on our results from the fluorescence substrate hydrolysis, we propose PAR‐3 as the additional regulator of IL‐8 and CXCL1 production. The PAR‐3‐based substrate is the most preferred by KLK14, being hydrolyzed ~4 times more efficiently in comparison to PAR‐2 and ‐4. In accordance with KLK14 substrate specificity (preference for Arg in the P1 position), the PAR‐3 receptor is cleaved at the noncanonical position R^41^/G^42^. Hydrolysis of the PAR‐3 receptor at this position has been described only for few proteinases, exemplified by activated protein C [45] and factor Xa [46]. This cleavage was found to result in the altered receptor signaling compared to the canonical thrombin cleavage at K^38^/T^39^. Indeed, our results from HSF stimulation by PAR agonist confirm the involvement of PAR‐3 in the induction of IL‐8 and CXCL1 production. As KLK14 shows preference toward the PAR‐1 activation site at Arg46, and biased PAR‐1 processing was implicated in the reduced inflammatory response by activated protein C and thrombin in the endothelium [44, 47, 48], we propose that similarly this alternate cleavage of PAR‐1 at Arg46 may be a limiting checkpoint of CXCL1 release, while, when PAR‐1 is blocked, activation of PAR‐3 takes over and impacts the CXCL1 release. Indeed, similar findings were described in the poly I:C‐induced airway inflammation in mice, where PAR1^−/−^ mice were characterized by increased CXCL1 levels in serum 24 h post‐treatment and the Arg46, not the Arg41 cleavage site was responsible for the limitation of the CXCL1 production [49]. Interestingly, KLK5 and KLK14 were previously shown to activate PAR2 expressed in keratinocytes and intense KLK14 staining was observed in atopic dermatitis and rosacea patients' squamous and granular skin layers [21]. Herein we identify KLK14 as a potent activator of PAR‐1 and, potentially, of PAR‐3. The epithelial skin layer is responsive to PAR‐2 activation, yet KLK14‐mediated PAR‐1/3 activation, described herein indicates the ability to stimulate the underlying dermal layer, resulting in the crosstalk between these skin compartments and an increase in inflammatory signaling. These findings suggest that KLK14 plays a central role in wound healing by regulating fibroblast secretion of proinflammatory cytokine levels. This process involves complex receptor activation, particularly of PAR‐1 and PAR‐3, which coordinate cytokine release and wound healing responses. Given the potential link to cancer progression, further investigation into KLK14's role in both wound healing and tumor microenvironments is warranted.

Harold Dvorak proposed an understanding of cancer as a never‐healing wound due to the similarities between the tumor stroma and the wound‐healing response [50]. Indeed, the local microenvironment may promote cancer progression by aberrant communication within the tissue [51, 52], and IL‐6, IL‐8, and CXCL1 were identified as the link between cancer and wound healing [25]. It was demonstrated that the tumor samples of squamous cell carcinoma (SCC) contained high levels of *IL‐6*, *IL‐8*, and *CXCL1* gene transcripts, a trait that was transferred to normal fibroblasts upon their co‐culture with FaDu carcinoma cells, suggesting paracrine signaling between these cells. Further, treating normal keratinocytes with IL‐6, IL‐8, and CXCL1 induced the expression of keratin‐8 in these cells, which is an indicator of poor prognosis in SCC patients [53]. Augmented expression and release of these molecular messengers in response to the KLK14 treatment tempt us to speculate about the nature of the long‐reported correlation between wound healing and cancer and hint at the need for a precise regulation of the KLK14 expression under normal conditions. Indeed, the activity of kallikreins is tightly controlled, and even under conditions where KLK activity is increased, it is often complemented by the augmentation of KLK‐targeting inhibitory molecule expression [54]. Despite novel results, our study has some important limitations. Most significantly, the cell–cell interactions were investigated in the individual 2D cultures. It is enticing to follow the presented research in the 3D system, more resembling the actual, differentiated skin, or in the tumor‐mimicking spheroid structures composed of both cell types, accompanied by full transcriptomics and proteomics analysis.

Herein we propose a model, where initial damage to the skin induces an increase in KLK14 extracellular activity, which in turn stimulates skin fibroblasts to release proinflammatory and chemotactic molecules, involved in the wound healing response. The modulation of IL‐6, IL‐8, and CXCL1 by KLK14 has potential implications beyond wound healing, particularly in cancer. Aberrant signaling between fibroblasts and keratinocytes in wound healing may mirror processes in cancer progression. Elevated levels of these cytokines have been associated with tumor growth and metastasis, and their regulation by KLK14 may represent a critical link between these processes. Further studies are needed to explore this potential connection.

## Materials and methods

### Cell culture

#### Cell culture reagents

Gibco® Dulbecco's Modified Eagle Medium 1× supplemented with 4.5 g·L^−1^ d‐Glucose and l‐Glutamine (DMEM), Gibco^®^ Fetal serum (FBS) and Gibco™ Penicillin–Streptomycin (10 000 U·mL^−1^) were purchased from Thermo Fisher Scientific (Life Technology, Warsaw, Poland). Phosphate‐buffered saline 10× (PBS) was purchased from Life Technologies and was diluted to 1× in distilled and autoclaved water. Trypsin–EDTA Solution 1× was purchased from Millipore‐Sigma (Warsaw, Poland).

#### Cell culture method

Human skin fibroblasts (HSF) were obtained in‐house by Dr Hab. Prof. UJ Justyna Drukała (Department of Cell Biology, Faculty of Biochemistry, Biophysics and Biotechnology, Jagiellonian University, Poland). Human skin fibroblasts (HSFs) were harvested from full‐thickness skin from healthy donors qualified for plastic surgery between June 2019 and September 2021. The skin came from plastic surgery performed at *Collegium Medicum*, Jagiellonian University, as a medical waste byproduct. The tissue samples were donated with formal ethical approval from the Jagiellonian University—Medical College Bioethics Committee (1072.6120.10.2017) in accordance with the standards set by the Declaration of Helsinki. The donors provided their written informed consent. Human HaCaT keratinocytes (HaCaT; RRiD: CVCL_0038) were purchased from the CSL Cell Lines Service GmbH. HSF and HaCaT cells were cultured in 10% FBS in DMEM supplemented with 0.5% Penicillin/Streptomycin in 75 cm^2^ tissue culture flasks (TPP, Switzerland) in 5% CO_2_ and 37 °C. Upon cell confluency, the cells were washed with 1× PBS and were detached with 2 mL of 1× trypsin–EDTA. Then, the conditioned media was returned to the flask and the cell suspension was centrifuged at 280 rcf for 8 min. The cell pellet was resuspended in fresh culture media and harvested for experimental purposes or split for further culturing. HSF were cultured for up to seven passages. Cell banks were routinely tested for mycoplasma presence every 6 months and were negative.

#### HSF cell stimulation

HSF were cultured on 24‐ or 12‐well cell culture plates (TPP, Switzerland) in DMEM supplemented with 10% (v/v) FBS and 100 U·mL^−1^ penicillin–streptomycin in standard conditions (5% CO_2_ at 37 °C) until cell confluency was reached. Subsequently, the cells were rinsed three times with 1× PBS, were stimulated with the protein of interest in DMEM and were incubated in standard conditions (5% CO_2_ at 37 °C). KLK14 and KLK14 Mut_Ser220Ala_ were purified in‐house as recombinant proteins as described in [16]. Heat‐inactivated KLK14 was prepared by incubating KLK14 at 90 °C for 25 min. KLK14's catalytically active concentration was assessed prior to each experiment. Tris/NaCl buffer is the KLK14 vehicle control and consists of 0.1 M Tris (BioShop, EPRO, Władysławowo, Poland), 150 mm NaCl (POCH), 5 mm EDTA (BioShop, EPRO), 0.05% Tween‐20 (Millipore‐Sigma), pH 7.5. The PAR‐1 agonist TFLLR‐NH2, the PAR‐2 agonist SLIGRL‐NH2, and PAR‐4 agonist AY‐NH2 were purchased from Tocris Bioscience and the PAR‐3 agonist PAR‐3 (1–6) amide (human) trifluoroacetate salt (H‐TFRGAP‐OH) was purchased from Bachem. The PAR antagonists RWJ 56110, FSLLRY‐NH2, and ML 354 were all purchased from Tocris, and HSF were stimulated with PAR antagonists in DMEM for 1 h in standard conditions prior to introducing KLK14.

### Cytokine assays

The levels of IL‐6, IL‐8, and CXCL1 in HSF conditioned media were assessed using commercially available enzyme‐linked immunosorbent assays (ELISAs) and were assayed according to the manufacturer's protocol. The IL‐6 and IL‐8 levels were measured using the Human IL‐6 ELISA Set BD OptEIA and the Human IL‐8 ELISA Set BD OptEIA purchased from BD Biosciences. CXCL1 levels were measured using the Human CXCL1/GRO alpha DuoSet ELISA from R&D Systems.

### Reverse transcription‐PCR

TRI reagent (Millipore‐Sigma) was used to extract cellular RNA according to the manufacturer's protocol. The RNA was reverse transcribed using the High Capacity cDNA Reverse Transcription Kit (Thermo Fisher Scientific). Each reaction consisted of 500 ng RNA in a total volume of 20 μL with random primers and the following conditions were applied: annealing at 25 °C for 10 min, extension at 37 °C for 120 min, reverse transcriptase inactivation at 85 °C for 5 min, and storage at −20 °C.

### Conventional PCR

PCR was performed utilizing the reverse transcribed cDNA and the DreamTaq Green PCR Master Mix (Thermo Fisher Scientific, Life Technology) according to the manufacturer's protocol. Each reaction mixture consisted of 25 ng cDNA, and the following conditions were utilized: Pre‐denaturation at 95 °C for 3 min, denaturation at 95 °C for 30 s, annealing at primer appropriate melting temperature (51.4 °C for PAR‐1, 46.2 °C for PAR‐2, 45 °C for PAR‐3, and 45.4 °C for PAR‐4) for 30 s, extension at 72 °C for 40 s, final extension at 72 °C for 5 min, storage at 4 °C, and the number of cycles was 35. Table 1 below demonstrates the primer sequences utilized.

**Table 1:** Primers used for conventional PCR.

Protein name	Primer	Sequence (5′‐3′)
PAR‐1	Forward	TACGCCTCTATCTTGCTCATGAC
	Reverse	TTTGTGGGTCCGAAGCAAAT
PAR‐2	Forward	CATCCTGCTAGCAGCCTC
	Reverse	TGACAGAGAGGAGGTCAG
PAR‐3	Forward	TTGTCAGAGTGGCATGGAA
	Reverse	ACAGGACCTCTCCAAATAC
PAR‐4	Forward	AACCTCTATGGTGCCTACGTGC
	Reverse	CCAAGCCCAGCTAATTTTTG

### Agarose gel electrophoresis

Agarose gel electrophoresis was performed to visualize the PCR products. A 1% agarose gel was prepared in 1× TAE buffer (40 mm Tris, 20 mm acetic acid, 1 mm EDTA, pH 8.5) and 0.5 μg·mL^−1^ of ethidium bromide (BioShop, EPRO). The PCR products were loaded to the gel and electrophoresis was performed for 30 min at 100 V. The gel was photographed under UV illumination Gel Doc™ EX Imager (Bio‐Rad Laboratories, Warsaw, Poland).

### Semi‐quantitative real‐time PCR

Semiquantitative real‐time PCR was conducted using the SYBR Green JumpStart Taq ReadyMix (Millipore‐Sigma). Each reaction consisted of a total volume of 15 μL with 1 μL (25 ng) of cDNA, 1× SYBR Green JumpStart Taq ReadyMix and 10 μL of each forward and reverse primers (Table 2). The reaction was initiated by pre‐denaturation at 95 °C for 5 min, followed by denaturation at 95 °C for 30 s, annealing at Tm (52 °C for *hCXCL1*, 56 °C for *hIL‐6*, 60 °C for hIL‐8, 56 and 60 °C for *hEF2*) for 1 min, extension at 72 °C for 1 min followed by the melt curve for a total of 41 cycles. The ‘delta–delta Ct’ quantification method [55] was used to calculate the results after obtaining the means for the threshold cycle values (Ct). The housekeeping gene elongation factor‐2 (*EF2*) was used for normalization as the reference.

**Table 2:** Primers used for semiquantitative real‐time PCR.

Oligonucleotide	Sequence (5′‐3′)
hCXCL1 F	AACCGAAGTCATAGCCACAC
hCXCL1 R	GTTGGATTTGTCACTGTTCAGC
hEF2 F	GACATCACCAAGGGTGTGCAG
hEF2 R	TTCAGCACACTGGCATAGAGGC
hIL‐6 F	AAATTCGGTACATCCTCGACGGCA
hIL‐6 R	AGTGCCTCTTTGCTGCTTTTCACAC
hIL‐8/hCXCL8 F	ATGACTTCCAAGCTGGCCGTGGCT
hIL‐8/hCXCL8 R	TCTCAGCCCTCTTCAAAAACTTCT

### Wound healing assay *in vitro*

#### HaCaT cytokine stimulation

Ibidi culture inserts were placed into the wells of 24‐well cell culture plates (TPP), and HaCaT cells were grown between the chambers of the inserts in standard conditions by seeding 0.25 million cells with a total of 500 μL·well^−1^ (70 μL between each insert chamber and 290 μL surrounding the insert) in DMEM supplemented with 2.5% (v/v) FBS and 0.5% (v/v) Penicillin/Streptomycin. After 24 h, the inserts were removed, displaying an open area; the wells were washed with 1× PBS, and the cells were stimulated with 0, 0.01, 0.05, 0.1, 0.5, 1, 5, or 10 ng·mL^−1^ of rhIL‐6, rhIL‐8, or rhCXCL1 (all from R&D Systems), respectively, in DMEM to a total volume of 500 μL·well^−1^. After a 72 h incubation in standard conditions, images were taken of the open area in each well using the Invitrogen™ Evos™ Fluorescence Microscope (Thermo Fisher Scientific, Life Technology) and a 10× objective lens. The CSE Lab TScratch Software tool was used to measure the open area in each image (https://github.com/cselab/TScratch).

#### HSF culture media transfer to HaCaT

Fully confluent HSF grown on 24‐well culture plates (TPP) were washed with 1× PBS and were stimulated with 200 nm KLK14 or 200 nm H.I. KLK14, respectively, in DMEM to a total volume of 400 μL·well^−1^. Subsequently, the plates were incubated for 24 h under standard conditions and the conditioned media were collected. Prior to further treatment, all cultured media were treated with 5 μm KLK14 inhibitor for 30 min at 37 °C (Biotin‐Tyr‐Gly‐Pro‐Arg‐CMK, in courtesy of Prof. Adam Lesner, Faculty of Chemistry, University of Gdansk, Poland).

Meanwhile, HaCaT were grown between the chambers of Ibidi 3‐well culture inserts as described above for 24 h. Subsequently, the inserts were removed, the cells were washed with 1× PBS and were treated with: 200 μL of the previously collected unstimulated control HSF conditioned media, 200 μL of unstimulated control HSF conditioned media + 25 ng·mL^−1^ human recombinant EGF (rhEGF) (Millipore‐Sigma), 200 μL of 200 nm KLK14 stimulated HSF conditioned media, 200 μL of 200 nm H.I. KLK14 HSF conditioned media, 200 μL of 200 nm KLK14 stimulated HSF conditioned media + 100 ng·mL^−1^ anti‐IL‐6 (Thermo Fisher Scientific, Life Technology), and 200 μL of unstimulated control HSF conditioned media + 100 ng·mL^−1^ anti‐IL6, respectively, in DMEM. The plates were incubated for 48 h in standard conditions, and images were taken of the open area in each well using the Invitrogen™ Evos™ Fluorescence Microscope (Thermo Fisher Scientific, Life Technology) and a 10× objective lens. The CSE Lab TScratch Software tool was used to measure the open area in each image (https://github.com/cselab/TScratch).

### PAR‐substrate cleavage analysis

A 96‐well flat black bottom microplate (Greiner AG) was prepared with 200 nm KLK14 in TNET (0.1 M Tris, 150 mm NaCl, 5 mm EDTA, 0.05% Tween‐20, pH 7.5) with 5% DMSO (BioShop) and with and without 10 μm of substrate representing the PAR‐1, PAR‐2, PAR‐3, or PAR‐4 N‐terminal exodomain where proteolytic cleavage results in receptor activation, in a total of 100 μL/well. The substrate sequences were as follows: PAR‐1: ACC‐G‐LDPR↓SFLL‐K(Dnp); PAR‐1 alternative: ACC‐GFLLR↓NPND‐K(Dnp); PAR‐2: ACC‐G‐SKGR↓SLIG‐K(Dnp); PAR‐3: ACC‐G‐LPIKTFR↓G‐K(Dnp); and PAR‐4: ACC‐G‐PAPR↓GYPG‐K(Dnp), respectively, with the expected cleavage site marked by an arrow. Substrates were custom‐synthesized as previously described [56]. A kinetic fluorescence measurement was taken at excitation and emission wavelengths of 350 nm and 460 nm, respectively, for 60 min at 37 °C with 15 s intervals utilizing the microplate reader SPECTRAMAX GEMINI EM (Molecular Devices, Wokingham, UK).

## Conflict of interest

The authors declare that they have no conflicts of interest with the contents of this article.

## Author contributions

TK: conceptualization; LS, EB, KF, MK, GB, AM, NZ, ED, JK, ML, NH, MP, KB: investigation; LS, EB, KF, MK, NH, TK: methodology; LS, KF: data curation; LS, EB, TK: writing—original draft; LS, EB, TK: visualization; LS, EB: formal analysis; EB, ML, TK: validation; EB, GB, JK, JD, ML, MP, KB, GD, JP, TK: supervision; EB, TK: project administration; KF, MK, GB, AM, NZ, ED, JK, JD, ML, MP, KB, GD, JP: writing—review & editing; NZ, JP, TK: funding acquisition; JK, JD, ML, NH, MP, KB, GD, JP, TK: resources.

## Data Availability

The data that support the findings of this study are openly available in RODBUK repository at https://doi.org/10.57903/UJ/QRCIOE.
